# Placental Expression of Sirtuins in Women with Gestational Diabetes

**DOI:** 10.3390/genes16070844

**Published:** 2025-07-20

**Authors:** Michał Czerewaty, Łukasz Ustianowski, Kajetan Kiełbowski, Estera Bakinowska, Krzysztof Safranow, Maciej Tarnowski, Tomasz Sroczyński, Andrzej Pawlik

**Affiliations:** 1 Department of Physiology, Pomeranian Medical University, 70-111 Szczecin, Poland; michal.czerewaty@wp.pl (M.C.); l.ustianowski@gmail.com (Ł.U.); kajetan.kielbowski@onet.pl (K.K.); esterabakinowska@gmail.com (E.B.); tomasz.sroczynski8@gmail.com (T.S.); 2 Department of Biochemistry and Medical Chemistry, Pomeranian Medical University, 70-111 Szczecin, Poland; chrissaf@mp.pl; 3 Department of Physiology in Health Sciences, Pomeranian Medical University, 70-210 Szczecin, Poland; m.tarnowski@interia.pl

**Keywords:** gestational diabetes mellitus, placenta, sirtuin

## Abstract

Background/Objectives: Gestational diabetes mellitus (GDM) is a common metabolic disorder in pregnant women. It can lead to several complications, such as preterm delivery, macrosomia, or metabolic disorders in newborns. Studies have revealed morphological and transcriptional differences between the placentas of patients with GDM and women with normal glucose tolerance. Sirtuins (SIRTs) are nicotinamide adenine dinucleotide-dependent deacetylases that interact with and regulate the activity of numerous proteins. However, little is known about their role in the pathogenesis of GDM. This study was performed to analyze the placental expression of SIRTs and investigate their correlations with clinical parameters. Methods: GDM was diagnosed based on the 75 g oral glucose tolerance test in accordance with the criteria developed by the International Association of Diabetes and Pregnancy Study Groups. Placental tissues were collected, and the expression of *SIRT1,-3,-4* and a reference gene (β-2 microglobulin) was analyzed. Results: The placental expression of *SIRT1* and *SIRT3* was elevated in women with GDM. However, there was no significant difference in *SIRT4* expression between women with GDM and those with normal glucose tolerance. Furthermore, we found no significant correlations between *SIRT1*, *SIRT3*, and *SIRT4* expression and clinical parameters. Conclusions: The findings of this study demonstrate elevated expression of *SIRT1* and *SIRT3* in the placentas of women with GDM. Further studies are required to confirm our observations and demonstrate the precise role of these enzymes in GDM.

## 1. Introduction

Many changes occur during pregnancy that make it easier to provide the nutrients and oxygen needed for the growing fetus. Because the fetus is dependent on maternal glucose delivery, these adaptations also involve glucose metabolism. For instance, the placenta secretes hormones such as human chorionic gonadotropin (hCG), human placental lactogen (hPL), and human placental growth hormone (hPGH) that may induce a state of insulin resistance [1]. Furthermore, changes in the efficacy of gluconeogenesis occur throughout pregnancy [1]. A state of hyperglycemia that is first recognized during pregnancy is known as gestational diabetes mellitus (GDM). According to the Diabetes Atlas of the International Diabetes Federation, the global prevalence of GDM in 2021 was 14% [2]. This metabolic disorder has been associated with several complications, including macrosomia, preterm delivery, and increased caesarean section rates, among others [3]. The pathogenesis of GDM is unclear; therefore, understanding the pathophysiological processes associated with GDM could result in the development of better treatment strategies.

Sirtuins (SIRTs) are nicotinamide adenine dinucleotide-dependent deacetylases that catalyze post-translational modifications of proteins. Seven members of the SIRT family (Sirtuin-1,-2,-3,-4,-5,-6,-7) have been identified in mammals, and the modifications that they perform involve deacetylation, ADP-ribosylation, and decrotonylation, among others [4]. SIRTs interact with numerous proteins, thus modulating their activity [5].

Consequently, their dysregulation has been suggested to play a role in the pathogenesis of various diseases. For instance, reduced placental expression of the *SIRT1* gene has been found in patients with preeclampsia [6]. However, the data regarding their role in the placentas of patients with GDM are limited. In this study, we examined the placental expression of *SIRT1*, *SIRT3*, and *SIRT4* in women with GDM and their correlations with clinical parameters.

## 2. Materials and Methods

### 2.1. Participants

This case–control study involved 26 women with GDM and 28 pregnant women with normal glucose tolerance. The clinical characteristics of women with GDM and women in the control group are shown in Table 1. The diagnosis of GDM was established in accordance with the criteria of the International Association of Diabetes and Pregnancy Study Groups [7]. A 75 g oral glucose tolerance test was performed, and GDM was diagnosed if the fasting glucose level was ≥92 mg/dL with 1- and 2 h plasma glucose concentrations of >180 and >153 mg/dL, respectively. The following clinical parameters were analyzed: age; fasting glucose concentration; daily insulin requirement; body mass index; body mass before pregnancy, at birth, and throughout pregnancy; newborn mass; and Apgar score. In the GDM cohort, 78% of patients were treated with diet alone and 22% were treated with diet and insulin until delivery. Insulin therapy was initiated if morning glycemia exceeded 95 mg/dL for 3 consecutive days despite an adequate diet or if the blood glucose level exceeded 140 mg/dL after a meal. The initial dose of insulin was 0.7 IU/kg body weight once daily, and it was adjusted according to the blood glucose concentration.

The study excluded women with acute or chronic complications such as diabetic ketoacidosis, as well as other disorders of glucose metabolism, autoimmune diseases, and chronic non-inflammatory diseases. Written consent for participation was obtained from all women, and the study was approved by the Ethics Committee of Pomeranian Medical University, Szczecin, Poland (KB-0012/40/14).

### 2.2. RNA Isolation

Placentas were collected from women who had undergone a natural delivery after 37 weeks of gestation at the Department of Obstetrics and Gynecology of Pomeranian Medical University. The tissues were transported to the Department of Physiology in a container with 0.9% NaCl solution. Approximately 100 mg of tissue was resected from the maternal side of the cotyledons for RNA extraction. No blood vessels, calcium deposits, or connective tissues were present in the study samples. Tissue samples were cut into small fragments and immediately stored in RNAlater^®^ Solution (Thermo Fisher Scientific, Waltham, MA, USA) at −80 °C until the time of genetic analysis. An RNeasy Mini Kit (Qiagen, Hilden, Germany) was used for RNA extraction in accordance with the manufacturer’s protocol. A DeNovix DS-11 FX spectrophotometer (DeNovix, Wilmington, DE, USA) was used to determine the RNA concentration and purity.

### 2.3. Real-Time Quantitative Reverse-Transcription Polymerase Chain Reaction

In total, 0.4 μg of RNA from each sample was reverse-transcribed into copy DNA (cDNA) in a total volume of 20 μL using the RevertAid First Strand cDNA Synthesis Kit (Thermo Fisher Scientific, Waltham, MA, USA) according to the manufacturer’s instructions. The quantitative expression of *SIRT1*, *SIRT3*, *SIRT4*, and a reference gene was analyzed using real-time quantitative reverse-transcription polymerase chain reaction on an ABI PRISM^®^ Fast 7500 Sequence Detection System (Applied Biosystems, Waltham, MA, USA), as previously described. The reference gene [β-2 microglobulin (*B2M*)] was determined based on the available literature [8,9,10]. A total of 2 μL of cDNA was present in each reaction. Two technical repeats were performed for each sample analysis, and the mean cycle threshold (Ct) values were used in further calculations. The final values were calculated with the comparative Ct method (2^−ΔCt^ method). The following primers were used for gene expression analysis:

*B2M* forward: 5′-AATGCGGCATCTTCAAACCT-3′
*B2M* reverse: 5′-TGACTTTGTCACAGCCCAAGA-3′
*SIRT1* forward: 5′-ACGCTGGAACAGGTTGCGGG-3′
*SIRT1* reverse: 5′-AGCGGTTCATCAGCTGGGCAC-3′
*SIRT3* forward: 5′-AAGTGTTGTTGGAAGTGGAG-3′
*SIRT3* reverse: 5′-TGTGAAAGAAGAATGGGAGT-3′
*SIRT4* forward: 5′-AGACTCCTTGTGATGACTGG-3′
*SIRT4* reverse: 5′-AGTACAGCTTTCCGAGTTTC-3′

### 2.4. Statistical Analysis

Statistical analysis was performed using STATISTICA version 13 (TIBCO Software, Inc., Palo Alto, CA, USA). Since the distributions of quantitative variables differed significantly from the normal distribution (Shapiro–Wilk test), non-parametric tests were used. Expression of *SIRTs* in the placenta was compared using the Mann–Whitney test. Spearman’s rank correlation coefficient (Rs) was used to examine the relationships between placental gene expression and clinical parameters.

Our study including 28 GDM women and 26 non-GDM controls had sufficient statistical power to detect with 80% probability true associations, corresponding to 0.78 of standard deviation of gene expression for difference between the groups and |Rs| > 0.5 for correlations within the GDM group. *p*-values of <0.05 were considered statistically significant. Results are presented as scatterplots where each dot represents one individual from the data set.

## 3. Results

**Figure 1:**
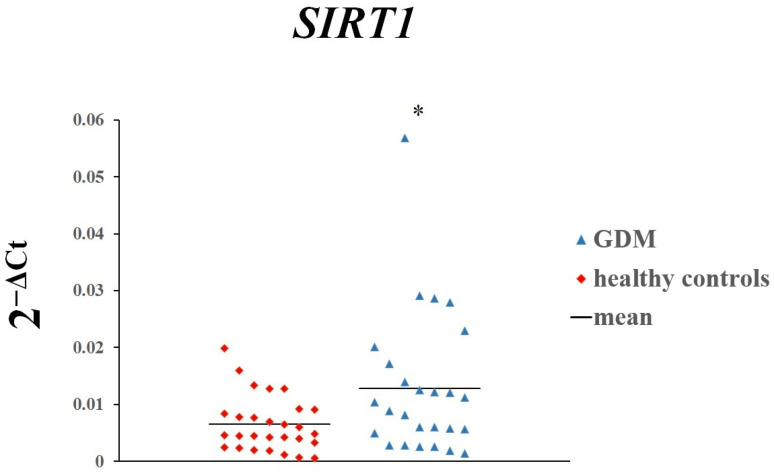
The expression of *SIRT1* in placenta of women with GDM and those with normal glucose tolerance. Each dot on the scatterplot represents one individual from the data set. * *p* < 0.05.

**Figure 2:**
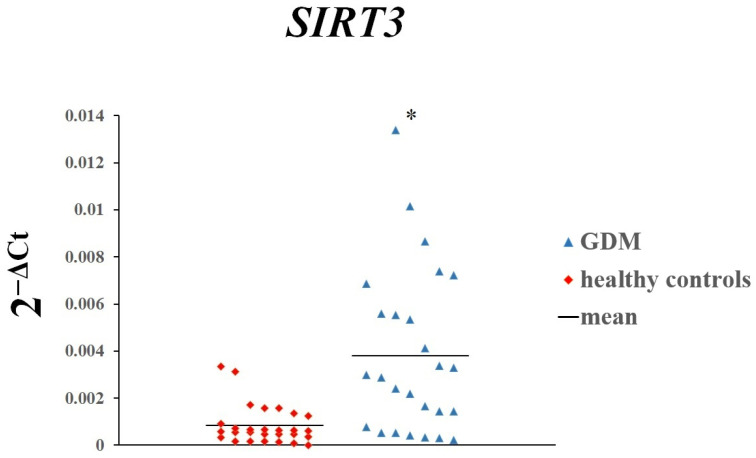
The expression of *SIRT3* in placenta of women with GDM and those with normal glucose tolerance. Each dot on the scatterplot represents one individual from the data set. * *p* < 0.05.

**Figure 3:**
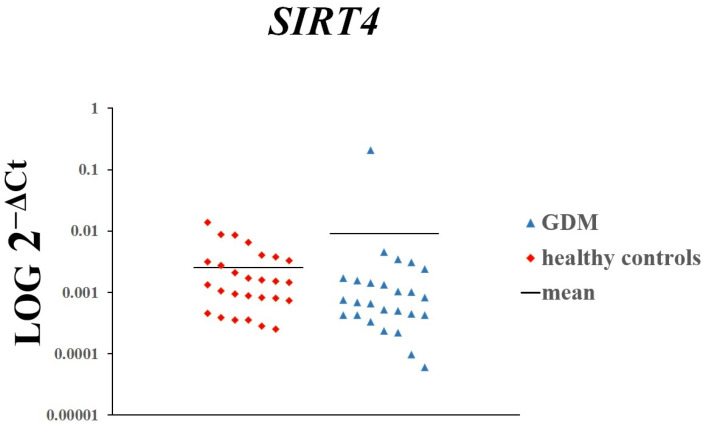
The expression of *SIRT4* in placenta of women with GDM and those with normal glucose tolerance. Each dot on the scatterplot represents one individual from the data set.

The placental expression of *SIRT1* (*p* = 0.01963) and *SIRT3* (*p* = 0.00008) was significantly higher in women with GDM than in women with normal glucose tolerance (Figure 1 and Figure 2). In contrast, there was no difference in the expression of *SIRT4* (Figure 3).

We also examined the correlations between placental expression of *SIRT1*, *SIRT3*, and *SIRT4* and selected clinical parameters in pregnant women with GDM. However, no significant correlations were found (Table 2, Table 3 and Table 4).

## 4. Discussion

GDM is a common metabolic disorder, and its pathophysiology remains unclear. Recent studies have demonstrated functional alterations and transcriptional differences in the placentas between patients with GDM and healthy controls [11]. Specifically, the placentas of patients with GDM show significantly greater villous immaturity than those of patients with type 1 and 2 diabetes [12]. In a recent study by Aldahmash et al. [13], placentas from patients with GDM showed several vasculopathies, including villous agglutination, retroplacental hemorrhage, calcification, and villous fibroid necrosis, among others. Furthermore, multiple differentially expressed genes between GDM and control placentas have been identified. Significantly upregulated genes (such as SLC1A6, ADRB1, and SLC1A2) may be involved in processes associated with antigen presentation and estrogen signaling [11].

Sirtuins are regulators of many cellular processes and metabolic pathways that occur in various tissues, such as skeletal muscle, pancreas, and liver. All of these tissues are involved in carbohydrate metabolism, tissue glucose transport, and the occurrence of insulin resistance [14]. Mitochondrial sirtuins are important regulators of cellular energy processes. Disruption of these processes can be one of the causes of impaired insulin production in the pancreas or the occurrence of insulin resistance in muscle tissue [4]. Also in pregnant women, sirtuins have important regulatory functions through which they regulate carbohydrate metabolism and may influence the development of GDM [15]. Sirtuins appear to be regulators that, at multiple levels and in various tissues, are responsible for controlling many metabolic processes, including those that may lead to the development of GDM. Previous studies indicate that sirtuins play an important role in the development of GDM, not only in the placenta of pregnant women, but also in tissues such as the pancreas, liver, and skeletal muscle [16]. Sirtuins can affect both insulin secretion and the development of insulin resistance.

Sirtuins regulate multiple signaling pathways that influence the development of inflammation, one of the causes of GDM [17,18]. Sirtuins regulate the NF-κB pathway, which plays an important role in inflammatory processes. They also influence TLR receptors, particularly TLR4, which play an important role in inflammatory responses in tissues, taking part in the secretion of pro-inflammatory cytokines and other mediators [19,20].

In this study, we found increased expression of placental *SIRT1* and *SIRT3* in women with GDM. These enzymes take part in major cellular mechanisms, and dysregulation of their expression has been associated with metabolic disorders. For instance, downregulation of SIRT1 was found in peripheral blood mononuclear cells of patients with metabolic syndrome and insulin resistance [21]. Conversely, overexpression of SIRT1 improves insulin sensitivity [22]. In addition, altered expression of SIRTs has been found in placental tissue of women with pregnancy complications, such as preeclampsia [6] and fetal growth restriction [23].

Members of the SIRT family are expressed in the placenta [24], and they have been suggested to take part in its physiological development. Deficiency of SIRT1 and SIRT3 is associated with various dysfunctions of trophoblasts [25,26]. In addition, overexpression of SIRT2 in trophoblasts improves their viability [27]. Nevertheless, the expression and role of SIRTs in the pathogenesis of GDM remain unclear. To date, there are only few papers that present often contradictory results regarding the role of sirtuins in the pathogenesis of GDM. Upregulation of *SIRT1* mRNA expression has been observed in leukocytes of patients with GDM [28]. In line with our findings, Zhang et al. [29] recently found elevated mRNA and protein expression of SIRT1 in placental tissue of patients with GDM. Intriguingly, elevated protein expression of PI3K/AKT was also observed. In subsequent analyses, the authors demonstrated that the placenta produced exosomes containing microRNA-135a-5p, which promotes SIRT1 and PI3K/AKT expression, suggesting that SIRT1 controls the pathogenesis of GDM [30]. The opposite results were observed by Han et al., who detected significantly lower levels of SIRT1 expression in the placental tissue and serum of their GDM group compared to controls [31].

SIRT1 regulates important processes of autophagy, senescence, and the response to oxidative stress in the placenta [32]. Autophagy is involved in the preservation of energy homeostasis, and studies have shown that this process is impaired in the placentas of patients with GDM [33,34]. SIRTs are deacetylases, and their altered expression may therefore result in abnormal profiles of acetylated proteins. Indeed, a recent study by Hu et al. [34] revealed numerous differently regulated acetylated proteins in placental tissues of patients with GDM. Furthermore, another study demonstrated that endothelial cells derived from umbilical cord tissue of patients with GDM had impaired antioxidant activity and showed senescent markers [35]. Interestingly, the authors found that the endothelial cells exerted reduced SIRT1 activity but elevated SIRT1 protein and mRNA expression. These results were accompanied by increased expression of acetyltransferase p300 and acetylated p53 [36]. Thus, the elevated expression of *SIRT1* and *SIRT3* observed in the present study could also represent the compensatory mechanisms. SIRT1 transcription is regulated by various transcription factors, including P53, as well as by negative feedback loops, causing SIRT1 expression to change depending on the metabolic state of cells [37,38,39,40,41].

Sirtuin 3 (SIRT3) is mainly found in mitochondria and is responsible for regulating metabolic processes, protecting cells from mitochondrial dysfunction and oxidative stress [42]. SIRT3 deficiency exacerbated hyperglycemia-induced mitochondrial damage and increased ROS accumulation [43,44]. SIRT3 is regulated by various mechanisms at the transcriptional and post-translational levels [45,46]. SIRT3 is a regulator of mitochondrial metabolism, and thus NAD+ and nicotinamide levels are direct regulators of its activity [47,48]. An important factor regulating the expression of both SIRT1 and SIRT3 is diet-related calorie availability [49,50,51,52,53,54]. The observed inter-population differences in sirtuin expression in the placenta of GDM women may be due to ethnic differences, the influence of diet and thus caloric availability, as well as factors affecting the transcriptional and post-transcriptional mechanisms regulating sirtuin expression [54,55,56].

Sirtuin 4 is a mitochondrial sirtuin that regulates the processes of lipid and carbohydrate metabolism [57]. The role of SIRT4 has not yet been extensively studied in the pathogenesis of GDM, but it has been shown that SIRT4 can also affect insulin secretion in pancreatic beta cells [58]. SIRT4 regulates adenine nucleotide translocase 2 (ANT2) activity and leucine catabolism, and thereby affects cellular ATP concentrations and pancreatic insulin secretion. Decreased expression of SIRT4 has been shown to activate NF-κB, resulting in increased inflammation. This also results in increased expression of cyclooxygenase-2 (COX2) and adhesion molecules, as well as increased synthesis of pro-inflammatory cytokines [59]. SIRT4 also inhibits the synthesis of apoptosis-increasing proteins such as p38 protein, Bax, and NADPH oxidases [59,60]. It was shown that fetal endothelial colony-forming cells (ECFCs) and human umbilical vein endothelial cells (HUVECs) from women with GDM had lower SIRT4 expression, which may result in the long-term cardiovascular complications observed in the offspring of pregnancies with GDM [61]. It has also been shown that in the early stages of diabetes mellitus type 2, SIRT4 levels may be reduced, which can result in the development of insulin resistance [14,62]. On the other hand, in advanced type 2 diabetes with its complications, an increase of SIRT4 in peripheral blood mononuclear cells was observed, which was explained by a feedback mechanism increasing SIRT4 levels [63].

Our study has several limitations. The main limitation of the study is the lack of determination of sirtuin proteins in placentas, as mRNA levels do not always correlate with protein expression, especially considering post-translational regulation. The study is also limited by the lack of histological evaluation of placental tissue.

The increased expression of SIRT1 and SIRT3 in the placenta shown in our study may suggest the involvement of these sirtuins in the pathogenesis of GDM. The pathogenesis of GDM is complex and involves a number of genetic, metabolic and environmental factors [46]. GDM is characterized by low-grade inflammation and infiltration of the placenta by immune cells, as well as metabolic changes in the placenta [47]. It is likely that some of the pro-inflammatory mediators or metabolites present in women with GDM may increase the expression of SIRT1 and SIRT3 in the placenta. However, understanding these mechanisms requires further research.

## 5. Conclusions

The present study revealed elevated expression of *SIRT1* and *SIRT3* in the placentas of patients with GDM. However, this elevated expression was not correlated with clinical parameters. Further research is required to confirm our observations and elucidate the role of SIRTs in the pathogenesis of GDM.

## Figures and Tables

**Table 1:** Clinical parameters in women with GDM and healthy women.

Parameters	GDM	Healthy Controls
	Mean ± SD	
Age (years)	32.3 ± 4.3	30.2 ± 4.8
Body mass before pregnancy (kg)	70.9 ± 16.3	69.4 ± 18.5
Body mass at birth (kg)	83.2 ± 15.7	81.1 ± 14.7
Body mass increase during pregnancy (kg)	12.3 ± 6.0	11.7 ± 7.6
BMI before pregnancy (kg/m^2^)	25.9 ± 5.5	24.7 ± 5.2
BMI at birth (kg/m^2^)	30.4 ± 5.4	29.1 ± 4.3
BMI increase during pregnancy (kg/m^2^)	4.5 ± 2.2	4.4 ± 2.6
Daily insulin requirement (unit)	13.7 ± 15.8	0 ± 0
Newborn body mass (g)	3401.9 ± 594.3	3318.6 ± 378.0
APGAR (0–10)	9.3 ± 0.8	9.5 ± 0.7

**Table 2:** Correlations between *SIRT1* expression in the placenta and clinical parameters in the GDM group.

Clinical Parameters	Rs	*p*
Age [years]	0.28	0.16
APGAR [0–10]	0.03	0.87
BMI at birth [kg/m^2^]	−0.05	0.81
BMI before pregnancy [kg/m^2^]	−0.05	0.80
BMI increase during pregnancy [kg/m^2^]	0.03	0.87
Body mass at birth [kg]	−0.08	0.71
Body mass before pregnancy [kg]	0.04	0.83
Body mass increase during pregnancy [kg]	0.08	0.70
Daily insulin requirement [unit]	0.19	0.33
Newborn body mass [g]	−0.04	0.86
R_s_—Spearman rank correlation coefficient.		

**Table 3:** Correlations between *SIRT3* expression in the placenta and clinical parameters in the GDM group.

Clinical Parameters	Rs	*p*
Age [years]	0.33	0.09
APGAR [0–10]	−0.21	0.30
BMI at birth [kg/m^2^]	−0.08	0.70
BMI before pregnancy [kg/m^2^]	−0.12	0.56
BMI increase during pregnancy [kg/m^2^]	0.08	0.71
Body mass at birth [kg]	−0.11	0.58
Body mass before pregnancy [kg]	−0.05	0.82
Body mass increase during pregnancy [kg]	0.11	0.57
Daily insulin requirement [unit]	0.15	0.46
Newborn body mass [g]	−0.08	0.69
R_s_—Spearman rank correlation coefficient.		

**Table 4:** Correlations between *SIRT4* expression in the placenta and clinical parameters in the GDM group.

Clinical Parameters	Rs	*p*
Age [years]	0.11	0.58
APGAR [0–10]	0.09	0.67
BMI at birth [kg/m^2^]	−0.20	0.31
BMI before pregnancy [kg/m^2^]	−0.21	0.30
BMI increase during pregnancy [kg/m^2^]	0.07	0.73
Body mass at birth [kg]	−0.25	0.21
Body mass before pregnancy [kg]	−0.17	0.41
Body mass increase during pregnancy [kg]	0.09	0.66
Daily insulin requirement [unit]	0.02	0.90
Newborn body mass [g]	0.04	0.85
R_s_—Spearman rank correlation coefficient.		

## Data Availability

Data are contained within the article.
